# The Passenger Domain of Bartonella bacilliformis BafA Promotes Endothelial Cell Angiogenesis via the VEGF Receptor Signaling Pathway

**DOI:** 10.1128/msphere.00081-22

**Published:** 2022-04-05

**Authors:** Kentaro Tsukamoto, Kayo Kumadaki, Kaoru Tatematsu, Natsumi Suzuki, Yohei Doi

**Affiliations:** a Department of Microbiology, Fujita Health University School of Medicine, Toyoake, Aichi, Japan; b Department of Infectious Diseases, Fujita Health University School of Medicine, Toyoake, Aichi, Japan; c Division of Infectious Diseases, University of Pittsburgh School of Medicine, Pittsburgh, Pennsylvania, USA; University of Kentucky

**Keywords:** *Bartonella*, autotransporter, angiogenesis, endothelial cell, VEGF receptor, BafA

## Abstract

Bartonella bacilliformis is a Gram-negative bacterial pathogen that provokes pathological angiogenesis and causes Carrion’s disease, a neglected tropical disease restricted to South America. Little is known about how *B. bacilliformis* facilitates vasoproliferation resulting in hemangioma in the skin in verruga peruana, the chronic phase of Carrion’s disease. Here, we demonstrate that *B. bacilliformis* extracellularly secrets a passenger domain of the autotransporter BafA exhibiting proangiogenic activity. The *B. bacilliformis*-derived BafA passenger domain (BafA*_Bba_*) increased the number of human umbilical endothelial cells (HUVECs) and promoted tube-like morphogenesis. Neutralizing antibody against BafA*_Bba_* detected the BafA derivatives from the culture supernatant of *B. bacilliformis* and inhibited the infection-mediated hyperproliferation of HUVECs. Moreover, stimulation with BafA*_Bba_* promoted phosphorylation of vascular endothelial growth factor receptor 2 (VEGFR2) and extracellular-signal-regulated kinase 1/2 in HUVECs. Suppression of VEGFR2 by anti-VEGFR2 antibody or RNA interference reduced the sensitivity of cells to BafA*_Bba_*. In addition, surface plasmon resonance analysis confirmed that BafA*_Bba_* directly interacts with VEGFR2 with lower affinity than VEGF or Bartonella henselae-derived BafA. These findings indicate that BafA*_Bba_* acts as a VEGFR2 agonist analogous to the previously identified B. henselae- and Bartonella quintana-derived BafA proteins despite the low sequence similarity. The identification of a proangiogenic factor produced by *B. bacilliformis* that directly stimulates endothelial cells provides an important insight into the pathophysiology of verruga peruana.

**IMPORTANCE** Bartonella bacilliformis causes life-threatening bacteremia or dermal eruption known as Carrion’s disease in South America. During infection, *B. bacilliformis* promotes endothelial cell proliferation and the angiogenic process, but the underlying molecular mechanism has not been well understood. We show that *B. bacilliformis* induces vasoproliferation and angiogenesis by producing the proangiogenic autotransporter BafA. As the cellular/molecular basis for angiogenesis, BafA stimulates the signaling pathway of vascular endothelial growth factor receptor 2 (VEGFR2). Identification of functional BafA protein from *B. bacilliformis* in addition to B. henselae and *B. quintana*, the causes of cat scratch disease and trench fever, raises the possibility that BafA is a common virulence factor for human-pathogenic *Bartonella*.

## INTRODUCTION

The members of the genus *Bartonella* are Gram-negative, fastidious, facultative intracellular bacteria which are widely present in a variety of mammalian hosts, including humans and domestic and wild animals, and are transmitted via arthropod vectors or direct contact (1, 2). The genus currently consists of more than 30 species, and some of them are causes of emerging and reemerging human diseases (3, 4). The three best-known diseases are cat scratch disease, caused by Bartonella henselae, trench fever, caused by *B. quintana*, and Carrion’s disease, caused by *B. bacilliformis* (5). B. henselae and *B. quintana* also cause bacillary angiomatosis and peliosis hepatis, in which the bacteria form vasoproliferative tumor-like lesions in immunocompromised hosts (6). Carrion’s disease, caused by *B. bacilliformis*, is a deadly, vector-borne endemic bartonellosis unique in Andean valleys of South America, including Peru, Ecuador, and Colombia. This illness is biphasic; i.e., *B. bacilliformis* infects human erythrocytes, causing serious acute hemolytic anemia known as Oroya fever, followed by chronic infection called verruga peruana, which is characterized by cutaneous vascular eruptions similar to bacillary angiomatosis (7).

Endothelial cells are hypothesized to be the primary niche for *Bartonella* to establish infection, where the bacterial cells replicate and subsequently translocate into the bloodstream (8, 9). In addition, *Bartonella* species possess properties to accelerate proliferation of vascular endothelial cells, which results in the angioproliferative lesions observed in bartonelloses such as bacillary angiomatosis and verruga peruana. In order to understand the infectivity and pathogenicity of *Bartonella*, it is imperative to clarify the interaction between the bacteria and vascular endothelial cells. The molecular basis of *Bartonella*-induced vascular endothelial proliferation has been best studied in B. henselae. B. henselae can trigger secretion of vascular endothelial growth factor (VEGF) from EA.hy 926 cells and express VEGF on hypoxia-inducible factor 1 activation in HeLa 229 cells (10–12). On the other hand, endothelial cells generally express the gene at low levels and are the recipients of VEGF secreted by other types of cells (13, 14). Mechanistically, therefore, B. henselae stimulates macrophages and epithelial cells to indirectly proliferate endothelial cells by paracrine VEGF signaling (12, 15). Furthermore, BepA, a type IV secretion system (T4SS) effector protein of B. henselae, mediates the antiapoptotic effect on endothelial cells and contributes indirectly to vasoproliferation by promoting cell survival (16). In comparison with B. henselae, how *B. bacilliformis* interacts with endothelial cells is poorly understood. The *B. bacilliformis* genome does not contain either T4SS or its effector genes, yet the organism clinically causes severe angiogenesis (17). It was previously reported that GroEL, a chaperon protein, may be associated with endothelial cell growth in *B. bacilliformis* (18). However, GroEL itself exhibits no mitogenic activity. Meanwhile, we recently identified a *Bartonella*-derived proangiogenic factor designated BafA from B. henselae and *B. quintana* (19). BafA is an autotransporter protein, and the gene encoding BafA is conserved in most *Bartonella* species, including *B. bacilliformis*.

We therefore speculated that *B. bacilliformis* may produce a similar autotransporter that stimulates endothelial proliferation and the angiogenic process. In this study, two BafA ortholog candidates were identified in the *B. bacilliformis* genome, and one of them was shown to possess proangiogenic activity. The newly identified autotransporter likely plays an important role in the angioproliferative manifestations of Carrion’s disease.

## RESULTS

### Mitogenic property of *B. bacilliformis*.

**FIG 1:**
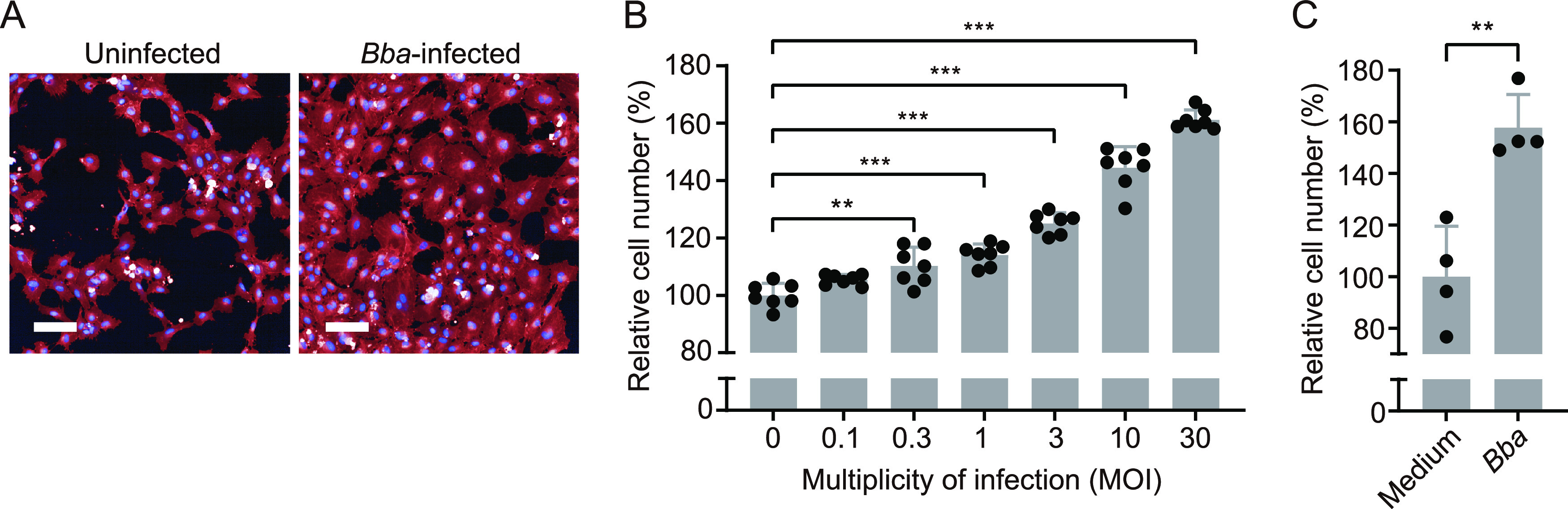
*B. bacilliformis* promotes vascular endothelial cell proliferation. (A) Fluorescence images of HUVECs cocultured with or without *B. bacilliformis* (*Bba*) at an MOI of 30 for 2 days. The cells were stained with CellMask deep red (red) and Hoechst 33342 (blue). Bar, 100 μm. (B) Infective dose-dependent cell-proliferative ability of *Bba*. The number of cells after infection at the indicated MOI was determined from the fluorescently stained images, and the values were normalized using the number of uninfected cells as 100%. (C) *Bba* promotes cell proliferation without bacterium-cell contact. The number of cells cocultured indirectly with *Bba* or with medium control is shown. Bars show means and SD (*n *= 8 or 4 biological replicates; circles). Statistical significance was determined using one-way ANOVA with Dunnett’s multiple-comparison test (**, *P < *0.01; ***, *P < *0.001).

Both whole cells and extracts of *B. bacilliformis* have been reported to promote endothelial cell proliferation (18, 20, 21). We first confirmed that *B. bacilliformis* is able to induce proliferation of human umbilical endothelial cells (HUVECs) as previously reported. As shown in Fig. 1A and B, *B. bacilliformis* promoted proliferation of HUVECs in proportion to multiplicity of infection (MOI), as reported in previous papers (19, 22). Moreover, cells cocultured with *B. bacilliformis* without direct bacterium-cell contact showed increased multiplication (Fig. 1C), confirming in this experiment that *B. bacilliformis* secretes a mitogen.

### Identification of a proangiogenic autotransporter of *B. bacilliformis*.

**FIG 2:**
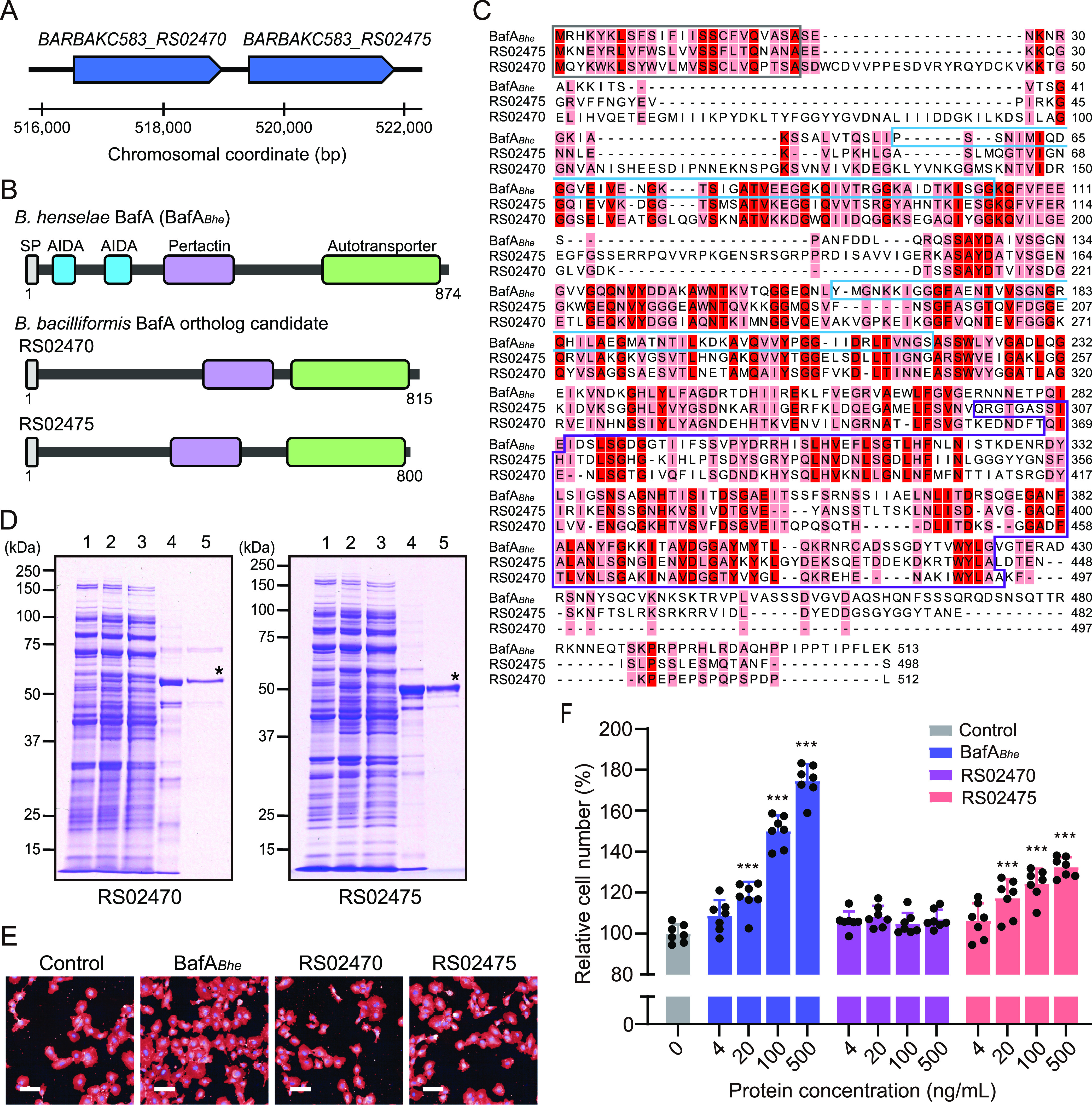
An autotransporter passenger domain of *B. bacilliformis* exhibits mitogenic activity. (A) Locus organization of *bafA* ortholog candidate genes (*BARBAKC583_RS02470* and *BARBAKC583_RS02475*) in the *B. bacilliformis* genome. Genes are drawn with chromosomal coordinates, and a scale bar is shown below the locus diagram. (B) Schematic representation of B. henselae-derived BafA (BafA*_Bhe_*) and its ortholog candidates from *B. bacilliformis* (RS02470 and RS02475). Conserved domains predicted by Pfam analyses are as follows: SP, signal peptide (gray); AIDA, adhesin involved in diffuse adherence (blue); pertactin, pertactin-like domain (purple); autotransporter, autotransporter β-domain (green). (C) Multiple-sequence alignment of the passenger domains. Conserved residues among the two sequences (light red) or three sequences (deep red) are highlighted. The domain architectures are surrounded by boxes: SP (gray), AIDA (blue), and pertactin (purple). (D) SDS-PAGE profiles of each purification step for the recombinant passenger domains. Asterisks indicate the proteins of interest in the final purification. Lanes: 1, uninduced whole cells of E. coli; 2, IPTG-induced whole cells of E. coli; 3, E. coli lysate; 4, eluate from immobilized Ni affinity chromatography; 5, eluate from size exclusion chromatography. (E) Fluorescence images of HUVECs treated with the autotransporter passenger domains. HBSS was used as a vehicle control. (F) Dose-dependent cell proliferation activity of the recombinant passenger domains. Bars show means and SD (*n *= 7 biological replicates; circles). Statistical significance was determined using one-way ANOVA with Dunnett’s multiple-comparison test compared with vehicle control (***, *P < *0.001).

We recently showed that B. henselae harbors the proangiogenic autotransporter BafA (19). The N-terminal passenger domain of BafA is extracellularly secreted and stimulates endothelial cell proliferation. We therefore speculated that *B. bacilliformis* may also produce a BafA ortholog(s) that plays a role in its mitogenic property. A BLAST search of the *B. bacilliformis* genome revealed the presence of two genes homologous to B. henselae *bafA* as ortholog candidates (Fig. 2A). Both proteins, encoded by *BARBAKC583_RS02470* (RS02470) and *BARBAKC583_RS02475* (RS02475), possessed predicted passenger domains containing a pertactin-like domain and a β-domain that matches the characteristics of Pfam “pertactin” (Pfam accession number PF03212) and “autotransporter” (PF03797). On the other hand, unlike the B. henselae-derived BafA (BafA*_Bhe_*), “AIDA” (PF16168) was not detected in the passenger domains of RS02470 and RS02475 (Fig. 2B). A multiple-sequence alignment showed that the deduced passenger domain of RS02470 shared 28.6% sequence identity with that of BafA*_Bhe_* with a 193-residue gap, whereas RS02475 shared 32.4% identity with BafA*_Bhe_* with an 87-residue gap and 25.9% identity with RS02470 with a 188-residue gap (Fig. 2C). Many conserved amino acid residues were found in the middle portions of the sequences (residues 221 to 493 in RS02470 or residues 154 to 442 in RS02475), but a number of gaps in the C-terminal regions were also identified, especially in RS02470.

To further verify which of the two candidates is the BafA ortholog, we performed synteny analysis of the genomic regions surrounding the *bafA* gene in B. henselae and *B. bacilliformis*. These regions are highly syntenic with each other (see Fig. S1 in the supplemental material). B. henselae harbored the *bafA* gene, tandemly located downstream of the two autotransporter genes (*BH05490* and *BH05500*), and the *pgsA* and *uvrC* genes further downstream. *B. bacilliformis* also had the conserved gene order, but only two autotransporter genes, *RS02470* and *RS02475*, were present in positions corresponding to the three autotransporter genes in B. henselae. Of these, *RS02475* was located downstream, suggesting that it was most likely the *bafA* ortholog. To assess the cell-proliferative activity of these *B. bacilliformis*-derived proteins, we generated recombinant RS02470 and RS02475 passenger domains. Both recombinant proteins were successfully obtained after size exclusion chromatography as almost single bands with a few minor bands detected on Coomassie brilliant blue-stained SDS-PAGE gels (Fig. 2D).

10.1128/msphere.00081-22.2 FIG S1 Comparison of genomic regions surrounding *bafA* between B. henselae and *B. bacilliformis*. The regions that show high-scoring segment pairs (LastZ score > 3,000) are connected with pink lines. Coding sequences are shown as green boxes, and *bafA* genes are shown as yellow boxes. Download FIG S1, PDF file, 0.2 MB. Copyright © 2022 Tsukamoto et al. 2022 Tsukamoto et al. https://creativecommons.org/licenses/by/4.0/ This content is distributed under the terms of the Creative Commons Attribution 4.0 International license.

**FIG 3:**
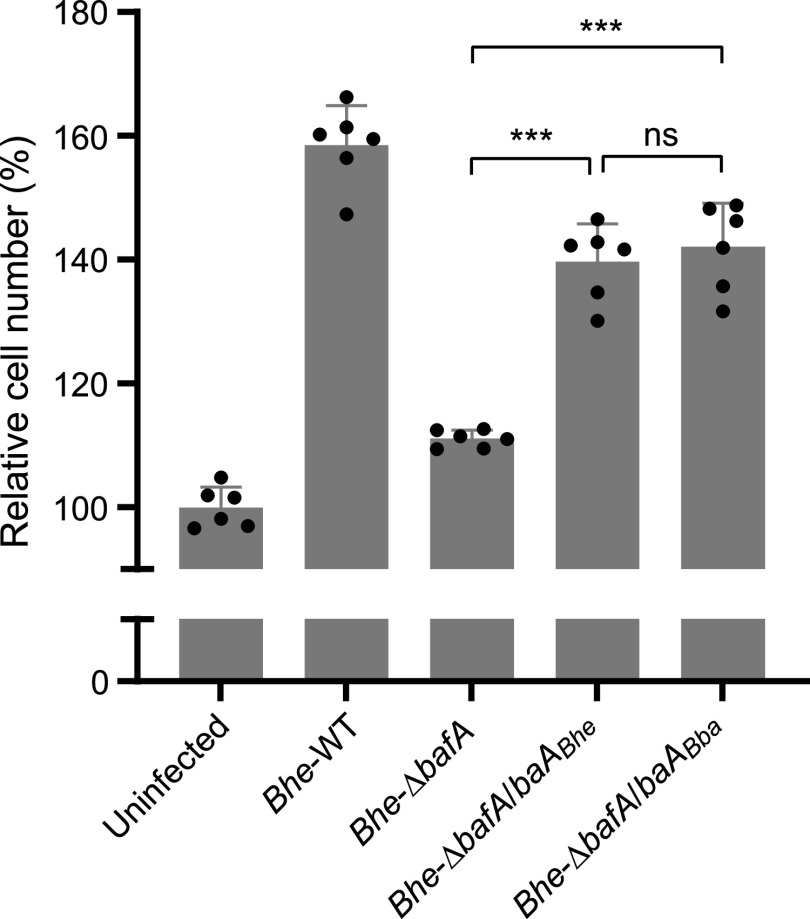
Complementation of *bafA_Bba_* recovers the mitogenic property of *bafA*-disrupted B. henselae. HUVECs were infected with indicated B. henselae mutants at an MOI of 30. *Bhe*-WT, B. henselae ATCC 49882; *Bhe*-Δ*bafA*, *bafA*-disrupted transposon mutant (B. henselae 623-125); *Bhe*-Δ*bafA*/*bafA_Bhe_*, 623-125 complemented with a *bafA_Bhe_*-carrying plasmid (19); *Bhe*-Δ*bafA*/*bafA_Bba_*, 623-125 complemented with a *bafA_Bba_*-carrying plasmid. Two days after infection, cell numbers were measured from HUVECs stained with CellMask deep red and Hoechst 33342. Bars show means and SD (*n *= 6 biological replicates; circles). Statistical significance was determined using one-way ANOVA with Tukey’s multiple-comparison test (ns, not significant; ***, *P < *0.001).

Next, we examined the mitogenic activity of the recombinant proteins in an endothelial cell proliferation assay. Extracellularly added RS02475 passenger domain increased the number of HUVECs, as has been observed with BafA*_Bhe_*, while the RS02470 passenger domain did not affect cell proliferation (Fig. 2E). The cell proliferation activity of RS02475 was dose dependent, but the number of cells after treatment with RS02475 was lower than that of cells treated with BafA*_Bhe_* (Fig. 2F). In order to further ascertain the functionality of BafA*_Bba_* on cell proliferation, we performed heterologous BafA complementation with a *bafA*-disrupted mutant of B. henselae. The B. henselae mutant transformed with the BafA*_Bba_* expression plasmid effectively restored the cell-proliferative property to the same extent as the mutant with homologous complementation (Fig. 3). From these observations, we consider RS02475 the *B. bacilliformis*-derived BafA ortholog; thus, we refer to it as BafA*_Bba_*.

**FIG 4:**
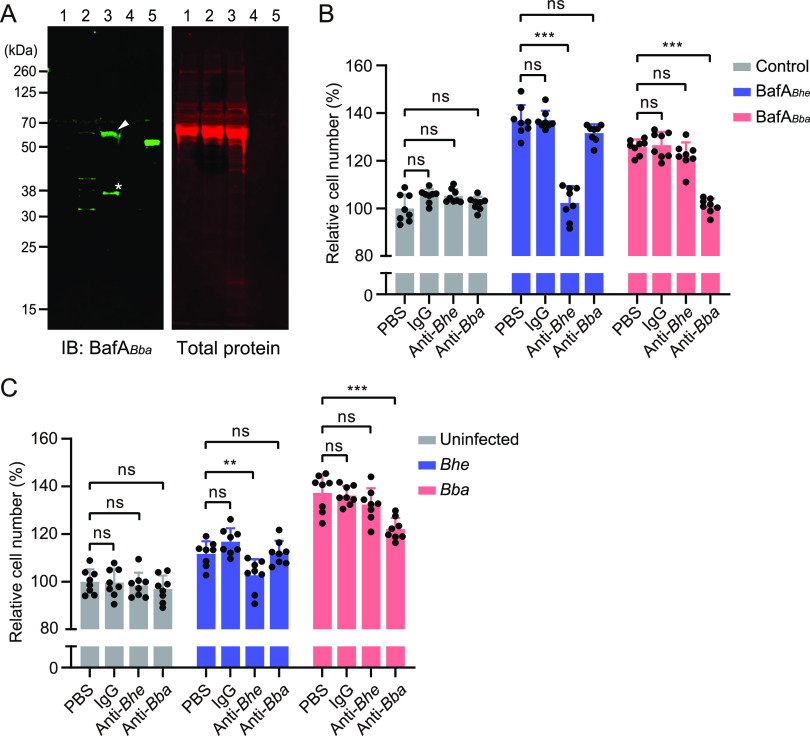
Inhibitory effect of anti-BafA antibodies on *B. bacilliformis*-induced cell proliferation. (A) Western blot analysis using anti-*B. bacilliformis*-derived BafA (BafA*_Bba_*) polyclonal antibody (left) and total-protein staining of the same membrane (right). An arrowhead indicates the BafA*_Bba_* passenger domain, and an asterisk represents its cleaved fragment. Lanes: 1, supernatant from HUVEC culture without bacteria; 2, supernatant with *Bhe*; 3, supernatant with *Bba*; 4, recombinant BafA*_Bhe_* passenger domain (3 ng/lane); 5, recombinant BafA*_Bba_* passenger domain (3 ng/lane). (B) Neutralization of BafA-induced cell proliferation with anti-BafA antibodies. HUVECs were pretreated with PBS, rabbit normal IgG, anti-BafA*_Bhe_* antibody, or anti-BafA*_Bba_* antibody (each at 15 μg/mL) before stimulation with vehicle control (HBSS), BafA*_Bhe_*, or BafA*_Bba_* (each at 100 ng/mL). (C) Neutralization of infection-mediated cell proliferation with anti-BafA antibodies. HUVECs were pretreated with PBS, rabbit normal IgG, anti-BafA*_Bhe_* antibody, or anti-BafA*_Bba_* antibody (each at 15 μg/mL) before infection with each species at MOI of 30. Bars show means and SD (*n *= 8 biological replicates; circles). Statistical significance was determined using one-way ANOVA with Dunnett’s multiple-comparison test (ns, not significant; **, *P < *0.01; ***, *P < *0.001).

We next investigated whether BafA*_Bba_* is actually secreted by *B. bacilliformis* and stimulates cell proliferation of endothelial cells. Using a Western blot analysis, two bands of approximately 60 kDa and 37 kDa that reacted with the anti-BafA*_Bba_* antibody were observed in the recovered fraction of the supernatant of *B. bacilliformis* (Fig. 4A). On the other hand, no specific bands were detected from the culture supernatant of uninfected HUVECs or cells cocultured with B. henselae. The proliferative activity of exogenously added BafA*_Bba_* was completely blocked by the pretreatment with anti-BafA*_Bba_* polyclonal antibody but not anti-BafA*_Bhe_* antibody (Fig. 4B). Moreover, the anti-BafA*_Bba_* antibody also decreased *B. bacilliformis*-induced cell proliferation in part, but no inhibitory effect of anti-BafA*_Bhe_* antibody was observed (Fig. 4C). These results suggest that BafA*_Bba_* plays a key mitogenic role in *B. bacilliformis*-induced endothelial cell proliferation.

**FIG 5:**
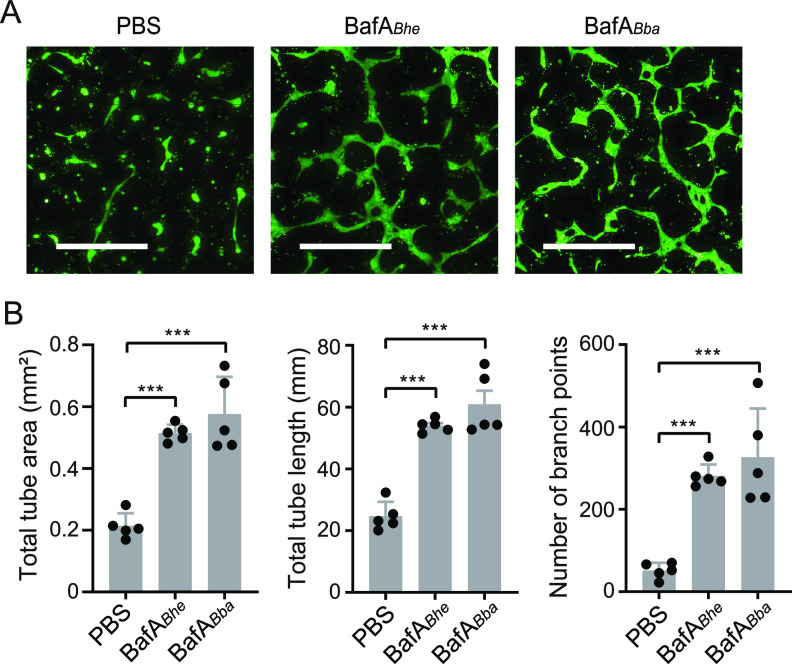
BafA*_Bba_* promotes *in vitro* endothelial tube formation. (A) Fluorescence images of tube-like structure of BafA (each at 100 ng/mL)-treated HUVECs stained with calcein AM. Bar, 500 μm. (B) Total tube area (left), total tube length (middle), and number of branch points (right) were calculated from the fluorescence images. Bars show means and SD (*n *= 5 biological replicates; circles). Statistical significance was determined using one-way ANOVA with Dunnett’s multiple-comparison test (***, *P < *0.001).

We then examined the proangiogenic activity of BafA*_Bba_* in a tube formation assay, an *in vitro* model of angiogenesis. In the presence of BafA*_Bba_*, HUVECs cultured for 20 h in type I collagen gels markedly formed tube-like structures, as has been observed with BafA*_Bhe_* (Fig. 5A). In reviewing the total tube area, length, and the number of branch points, which are indicators of tube formation, each value is comparable between BafA*_Bhe_* and BafA*_Bba_* (Fig. 5B), indicating that BafA*_Bba_* is capable of stimulating the angiogenic processes in endothelial cells.

### *B. bacilliformis*-derived BafA upregulates the VEGFR2 signaling pathway.

**FIG 6:**
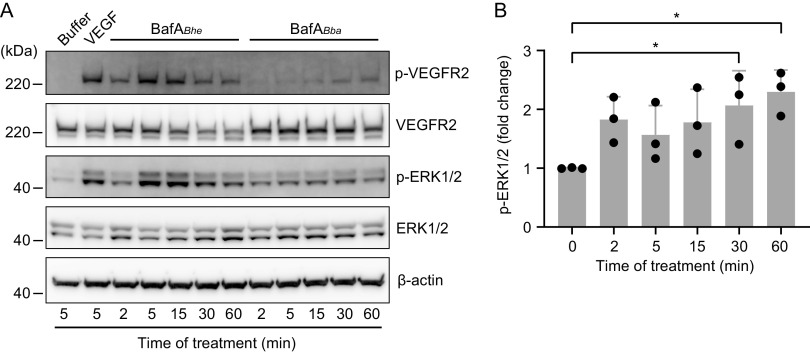
BafA*_Bba_* upregulates the VEGFR2-ERK signaling pathway. (A) Phosphorylation of VEGFR2 and ERK1/2 in HUVECs stimulated with vehicle control (HBSS), VEGF-A_165_ (20 ng/mL), BafA*_Bhe_* (200 ng/mL), or BafA*_Bba_* (200 ng/mL) for the indicated times. Western blot analysis was performed using antibody against phosphorylated (p-) and total VEGFR2 or ERK1/2. (B) Quantification of BafA*_Bba_*-induced phosphorylation of ERK1/2. The phosphorylation ratio was normalized to total ERK1/2, with the value for untreated cells (time zero) set as 1. Bars show means and SD (*n *= 3 biological replicates; circles). Statistical significance was determined using one-way ANOVA with Dunnett’s multiple-comparison test (*, *P < *0.05).

BafA*_Bhe_* has been shown to interact with VEGF receptor 2 (VEGFR2) and subsequently activate the downstream p44/p42 (ERK1/2) mitogen-activated protein kinase (MAPK) signaling pathway in endothelial cells (19). We assessed the effect of BafA*_Bba_* on this pathway by Western blot analysis. During the 60-min treatment with BafA*_Bba_*, the detectable signals of phosphorylated VEGFR2 were elevated over time, although the levels with BafA*_Bba_* were lower than those with VEGF-A_165_ or BafA*_Bhe_* (Fig. 6A). Likewise, BafA*_Bba_* increased the phosphorylation level of ERK1/2 especially after 30 and 60 min of treatment (Fig. 6A and B). These observations suggest that BafA*_Bba_* functions as a VEGFR2 ligand in a manner similar to that of VEGF-A_165_ and BafA*_Bhe_*.

### BafA*_Bba_*-induced cell proliferation requires binding to VEGFR2.

**FIG 7:**
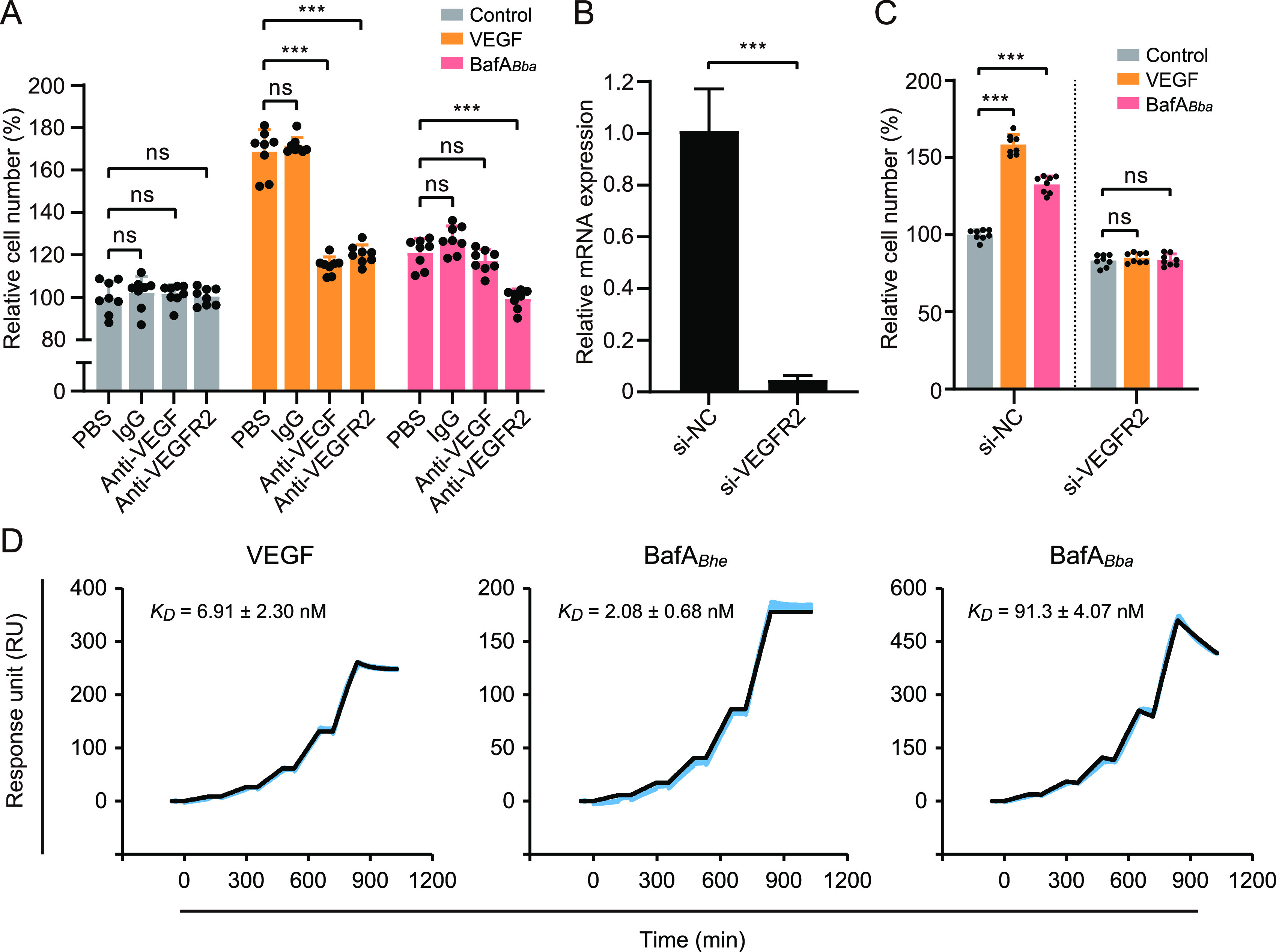
Effect of VEGFR2 inhibition on BafA*_Bba_* activity and binding profiles of VEGFR2 with BafAs. (A) Inhibitory effect of anti-VEGFR2 antibody on the mitogenic activity of BafA*_Bba_*. HUVECs were pretreated with PBS, human normal IgG (3 μg/mL), anti-VEGF antibody (bevacizumab, 3 μg/mL), or anti-VEGFR2 antibody (ramucirumab, 3 μg/mL), before stimulation with vehicle control (HBSS), VEGF-A_165_ (20 ng/mL), or BafA*_Bba_* (50 ng/mL). Bars show means and SD (*n *= 8 biological replicates; circles). (B) Expression of VEGFR2 in HUVECs transfected with noncoding small interfering RNA (si-NC) or siRNAs coding for VEGFR2 (si-VEGFR2). VEGFR2 mRNA relative expression was quantified by qRT-PCR. Bars show means and SD (*n *= 3 biological replicates). (C) Cell proliferation of siRNA-transfected HUVECs stimulated with control (HBSS), VEGF-A_165_ (20 ng/mL), or BafA*_Bba_* (100 ng/mL). Bars show means and SD (*n *= 8 biological replicates; circles). (D) Binding profiles of VEGF-A_165_, BafA*_Bhe_*, and BafA*_Bba_* with VEGFR2, characterized by surface plasmon resonance analysis. The Fc-tagged VEGFR2 was captured on protein A sensor chips and subsequently tested for binding with gradient concentrations (1.25, 2.5, 5, 10, and 20 nM) of VEGF-A_165_, BafA*_Bhe_*, or BafA*_Bba_* with the binding profiles shown. The *K_D_* values shown are means and SD from three independent experiments. Statistical significance was determined using one-way ANOVA with Dunnett’s multiple-comparison test (A and C) or a two-tailed unpaired Student's *t* test (B). ns, not significant; ***, *P < *0.001.

In order to determine whether BafA*_Bba_* exhibits mitogenic activity through direct interaction with VEGFR2, we next examined the effect of VEGFR2 blocking or silencing on BafA-induced HUVEC proliferation. Pretreatment with anti-VEGF antibody, which efficiently neutralizes VEGF-A_165_ activity, showed no effect on BafA-induced cell proliferation, whereas anti-VEGFR2 antibody effectively inhibited the BafA*_Bba_* activity (Fig. 7A). Furthermore, knockdown of VEGFR2 in HUVECs (Fig. 7B) completely abolished the potency of BafA*_Bba_* and VEGF-A_165_ stimulation (Fig. 7C). These results strongly suggested that BafA did not induce VEGF secretion from HUVECs but bound directly to VEGFR2, and we thus attempted to detect the interaction by surface plasmon resonance (SPR) assays. The recombinant protein of Fc-tagged VEGFR2 extracellular domain (VEGFR2-ECD) was captured on protein A-immobilized sensor chips and tested for binding with gradual concentrations of VEGF-A_165_, BafA*_Bhe_*, and BafA*_Bba_*. All three analytes were found to readily interact with VEGFR2-ECD (Fig. 7D). The equilibrium dissociation constants (binding affinity [*K_D_*]) of VEGF-A_165_ and BafA*_Bhe_* binding to VEGFR2-ECD were calculated to be 6.91 ± 2.30 nM and 2.08 ± 0.68 nM, respectively. The *K_D_* value of BafA*_Bba_*, which was determined to be 91.3 ± 4.07 nM, represented significantly lower binding affinity than that observed for VEGF-A_165_ and BafA*_Bhe_*. Taken together, these data indicate that the interaction between VEGFR2-ECD and BafA*_Bba_* exhibits 44-fold lower binding affinity than that with BafA*_Bhe_*.

## DISCUSSION

*B. bacilliformis* is a bacterial pathogen that is transmitted to humans by the bite of *Lutzomyia* sand flies, whose habitat is restricted to high-altitude valleys in the Andes Mountains of South America (23). Humans are the only known reservoir of *B. bacilliformis*, and infection results in Carrion's disease, a life-threatening bartonellosis. In the acute phase of Carrion's disease, known as Oroya fever, the bacteria infect erythrocytes and cause severe hemolytic anemia. Erythrocyte adherence and invasion involves several identified virulence factors, including flagella (24), the extracellular protein deformin (25), and invasion-associated locus proteins A and B (26). On the other hand, in verruga peruana, the chronic phase of Carrion disease, the bacteria infect endothelial cells and cause their proliferation, resulting in dermal eruption. The presence of numerous microvessels in the skin lesions suggests that infection of endothelial cells triggers a local angiogenic response. This fact was experimentally demonstrated in studies showing that *B. bacilliformis* enhanced endothelial cell proliferation and that several factors were involved in this process. One of these factors was tissue plasminogen activator (t-PA), production of which was elevated in infected HUVECs and might be involved in the angiogenic process (21). As a bacterium-derived factor, GroEL, a heat shock protein of *B. bacilliformis*, was reported to play an important role in inducing endothelial cell proliferation (18). GroEL was secreted from the culture supernatant of *B. bacilliformis* and exhibited an increase in the number of HUVECs in a dose-dependent manner, and this cell proliferation was partially inhibited in the presence of anti-GroEL antibody. However, mitogenic activity in HUVEC cultures treated with the recombinant GroEL protein was not detected, and the authors therefore suggested that the effect of GroEL on HUVECs might be indirect and might involve other factors. Thus, the mechanism by which *B. bacilliformis* facilitates endothelial cell proliferation still remains poorly characterized.

In this study, we successfully identified a proangiogenic autotransporter from *B. bacilliformis* that directly stimulates vascular endothelial cells. The passenger domain of this autotransporter is considered a BafA ortholog, since this protein displayed enhancement of endothelial proliferation, capillary formation, and upregulation of VEGFR2 signaling despite having only 32.4% amino acid sequence identity with that derived from B. henselae. Pertactin-like domains are present in the passenger of BafA*_Bba_* as well as those derived from B. henselae and *B. quintana*; therefore, they presumably represent a conserved domain structure for the proangiogenic autotransporters of *Bartonella*. On the other hand, another autotransporter RS02470 that showed 28.6% sequence identity with BafA*_Bhe_* also contained a pertactin-like domain but lacked cell-proliferative activity. Therefore, we speculate that the pertactin-like domain is necessary, but not sufficient, for the proangiogenic activity of the BafA family autotransporter.

For endothelial cell proliferation and angiogenesis, activation of ERK1/2 in endothelial cells is one of the most important signal transduction pathways (27, 28). BafA*_Bba_* evoked a dose-dependent increase in the number of HUVECs, but the percent increase of cell growth at higher concentrations of BafA*_Bba_* (100 or 500 ng/mL) was lower than that with BafA*_Bhe_*. In addition, relatively low phosphorylation levels of both VEGFR2 and ERK1/2 were detected in BafA*_Bba_*-treated cells compared to BafA*_Bhe_*-treated ones, suggesting that BafA*_Bba_* is modestly active in enhancing cell proliferation via upregulation of VEGFR2 signaling. The weak activity of BafA*_Bba_* is presumably attributed to its lower binding affinity for VEGFR2 than that of VEGF-A_165_ or BafA*_Bhe_*, but blocking or silencing of VEGFR2 abolished the responsiveness of HUVECs to BafA*_Bba_*, suggesting that the direct binding to VEGFR2 is an essential event for BafA*_Bba_* to upregulate the downstream signals.

Recombinant BafA*_Bba_* exhibits a rather marginal mitogenic activity compared to the effect observed with BafA*_Bba_* on the infected cells. One possible explanation is that the purified recombinant BafA*_Bba_* was different in size from both protein fragments (approximately 60 kDa and 37 kDa) detected by Western blot analysis when using *B. bacilliformis* culture supernatant. This suggests that the native BafA secreted from *B. bacilliformis* may be processed at a different position(s) than the recombinant we prepared. The mismatch of the size of BafA*_Bba_* may have affected the activity. Another possibility is that BafA*_Bba_* is unstable, and its activity was reduced during preparation, perhaps during extraction of protein from E. coli cells by ultrasonication or the subsequent purification steps. In fact, when the BafA-disrupted mutant of B. henselae was heterologously complemented with BafA*_Bba_*, it showed the same level of cell proliferation activity as when it was complemented with BafA*_Bhe_*. This supports the possibility that BafA*_Bba_* is intrinsically as active as BafA*_Bhe_*.

In contrast to the weak cell proliferation activity, BafA*_Bba_* facilitated the tube formation of HUVEC cultured in collagen gel at levels comparable to BafA*_Bhe_*. For capillary morphogenesis, the expression of integrins α_1_β_1_ and α_2_β_1_ has been elevated in the cell surface, and activation of Src kinase and Rho GTPase is a key intracellular event involved in tube morphogenesis (29–31). Moreover, activation of p38 MAPK also has been implicated as crucial in driving tubulogenesis of endothelial cells overlaid with type I collagen in a serum-free defined medium (32). It may be speculated that this tubulogenesis-related signaling with BafA*_Bba_* stimulation occurs at the same level as with BafA*_Bhe_*.

A polyclonal antibody against BafA*_Bhe_* displayed no inhibitory effect on the activity of BafA*_Bba_*, and conversely, anti-BafA*_Bba_* antibody was unable to suppress the BafA*_Bhe_* activity, indicating that these BafA proteins consist of distinct antigenicity structures. Neutralizing antibody against BafA*_Bba_* incompletely blocked the HUVEC proliferation during *B. bacilliformis* infection. This suggests that other factors such as t-PA and GroEL are also involved in the proangiogenic capability of *B. bacilliformis*. In addition, recent studies reported that infection of HUVECs by live *B. bacilliformis* induced host cell secretion of epidermal growth factor (EGF) (33). EGF is a growth factor associated with angiogenesis of vascular endothelial cells, and EGF levels in serum samples showed moderate positive correlation with *B. bacilliformis* bacteremia of Carrion’s disease in Peru (34). Collectively, multiple factors, including BafA, likely contribute to the hyperproliferation of endothelial cells and the subsequent angiogenic process in response to *B. bacilliformis* infection.

In summary, we identified a novel mechanism whereby *B. bacilliformis* provokes endothelial cell proliferation and angiogenesis by producing the proangiogenic autotransporter BafA during infection. A previous study reported that tyrosine kinase inhibitors decreased the invasion of endothelial cells by *B. bacilliformis* (35). In addition to serving as a mitogenic factor that promotes angiogenesis, BafA may therefore be involved in the internalization of *B. bacilliformis* into endothelial cells, as BafA can upregulate the tyrosine kinase activity of VEGFR2 and its downstream signaling molecules. We believe that BafA functions as a crucial pathogenic factor that gives rise to pathological angiogenesis and contributes to infection of humans by *B. bacilliformis*. The genomic regions around the *bafA* gene are highly conserved between B. henselae and *B. bacilliformis*. In addition to this finding, the presence of *bafA* orthologs in many *Bartonella* species (19) indicates that *bafA* is one of the most characteristic genes in bartonellae which were inherited from the ancestral species. Based on these findings, the BafA protein family is likely to be a bacterial apparatus common to *Bartonella* species that facilitates their survival and proliferation in hosts.

## MATERIALS AND METHODS

### Bacterial culture.

*B. bacilliformis* strain KC584 (ATCC 35686) was obtained from the American Type Culture Collection (Manassas, VA). *B. bacilliformis* was grown on Columbia agar with 5% defibrinated sheep blood (CSB; Becton Dickinson, Franklin Lakes, NJ) at 28°C. After 10 to 14 days of culture, the bacteria were collected from the culture plate and resuspended in medium 199 (M199; Thermo Fisher Scientific, Waltham, MA) supplemented with 10% fetal bovine serum (FBS; Biowest, France). For convenience, the bacterial number in the suspension was estimated to be 1 × 10^9^ bacteria/mL at an optical density at 600 nm (OD_600_) of 1.0. The details of the generation of heterologous BafA-complemented B. henselae are described in Text S1 in the supplemental material. Escherichia coli strains were grown on a lysogenic broth (LB) agar or in the liquid medium (Becton Dickinson). When required, kanamycin was used at a final concentration of 25 μg/mL.

10.1128/msphere.00081-22.1 TEXT S1 Supplemental methods. Details of the generation of heterologous BafA-complemented B. henselae and synteny analysis. Download Text S1, DOCX file, 0.02 MB. Copyright © 2022 Tsukamoto et al. 2022 Tsukamoto et al. https://creativecommons.org/licenses/by/4.0/ This content is distributed under the terms of the Creative Commons Attribution 4.0 International license.

### Endothelial cell proliferation assay.

HUVECs purchased from PromoCell (Heidelberg, Germany) were cultured in endothelial growth medium 2 (EGM-2; PromoCell) at 37°C in a humidified atmosphere and 5% CO_2_. For evaluation of proliferation of infected cells, HUVECs (passage 6 to 9, 9,000 cells/cm^2^) were plated onto a gelatin-coated 96-well plate with EGM-2. After 6 to 8 h of incubation, the medium was replaced with M199–10% FBS, and then the cells were infected with *B. bacilliformis* at the indicated MOI. For evaluation of the activity of BafA proteins, HUVECs were treated with indicated concentrations of BafA after changing the culture medium to M199–10% FBS. When the effect of antibodies was examined, anti-BafA*_Bhe_* antibody (15 μg/mL; generated in our previous study [19]), anti-BafA*_Bba_* antibody (15 μg/mL), anti-VEGF antibody (bevacizumab; Pfizer, 3 μg/mL), anti-VEGFR2 antibody (ramucirumab; Eli Lilly Japan; 3 μg/mL), normal rabbit IgG (15 μg/mL; Fujifilm Wako, Osaka, Japan), or human normal IgG (3 μg/mL; Fujifilm Wako) was used to treat cells for 30 min at 37°C prior to bacterial infection or addition of BafA or VEGF-A_165_ (PeproTech, Cranbury, NJ). Two days after bacterial infection or BafA treatment, the cells were stained with CellMask deep red plasma membrane stain (1:5,000; Thermo Fisher Scientific) and NucBlue Live ReadyProbes reagent (Thermo Fisher Scientific) for 30 min at 37°C. The cells were then fixed with 4% paraformaldehyde for 15 min at room temperature and washed with phosphate-buffered saline (PBS) three times. The plate was imaged on an Opera Phenix high-content screening system (PerkinElmer, Waltham, MA) using the confocal setting with a 5× or 20× air objective. Cell numbers were measured from images of 4 (with the 5× objective) or 25 (with the 20× objective) fields in each well using Harmony 4.5 software (PerkinElmer).

### Indirect coculture of HUVEC and *B. bacilliformis*.

To coculture HUVECs with *B. bacilliformis* without direct contact, a Millicell 24-well cell culture plate (Merck Millipore, Burlington, MA) was used to separate them as previously described (22). *B. bacilliformis* cells cultivated on a CSB plate for 14 days were collected and suspended in M199/10% FBS to an OD_600_ of 0.03. Two hundred microliters of the suspended *B. bacilliformis* cells was then added to filter plate wells, which were inserted into a receiver plate in which HUVECs were seeded at a density of 20,000 cells/well in M199–10% FBS. After 2 days in coculture, the Millicell filter plate was removed, and the proliferation of HUVECs was assessed as described under “Endothelial cell proliferation assay”.

### Bioinformatic prediction of BafA orthologs.

The deduced BafA orthologs in the *B. bacilliformis* genome (NCBI accession number NC_008783.1) were searched against the sequence of the passenger domain of B. henselae-derived BafA autotransporter using local BLAST program in CLC Main Workbench 21.0.3 software (Qiagen, Hilden, Germany) with an *E* value cutoff of <1 × 10^−50^. Conserved domain analyses in these candidates were conducted using the Pfam 34.0 server (http://pfam.xfam.org/). Signal peptides were predicted by the SignalP-5.0 server (https://services.healthtech.dtu.dk/service.php?SignalP-5.0). The passenger domains of BafA*_Bhe_*, RS02470, and RS02475 were aligned and compared by CLC Main Workbench. The details of synteny analysis are described in Text S1 in the supplemental material.

### Plasmid construction.

Primers used in this study were obtained from Thermo Fisher Scientific. The plasmid vector for expression of BafA*_Bhe_* (pET-28b-BH513) was generated in our previous study (19). For construction of plasmids expressing the passenger domain of *B. bacilliformis* BafA ortholog candidates, the genomic DNA of *B. bacilliformis* was purified from 14-day-old cultures on CSB agar using a PureLink genomic DNA mini kit (Thermo Fisher Scientific). Using the genomic DNA as the template, the nucleotides encoding the 25th to 512th amino acids of *BARBAKC583_RS02470* and the 25th to 498th amino acids of *BARBAKC583_RS02475* were amplified by Platinum SuperFi DNA polymerase (Thermo Fisher Scientific) with the primer sets NheI-RS02470-Fw (TCTCGCTAGCTCAGATTGGTGTGATGTCGTG)–SalI-RS02470-Rv (GACTGTCGACTCATAAAGGATCTGGACTTGGCTGA) and NheI-RS02475-Fw (TCTCGCTAGCGAGGAAAAAAAGCAAGGGGG)–SalI-RS02475-Rv (GACTGTCGACTCATGAAAAATTAGCAGTCTGCATACTC), respectively. The PCR products were digested with NheI/SalI and inserted into the compatible sites of pET-28b (Sigma-Aldrich, St. Louis, MO) by using a Ligation-Convenience kit (Nippon Gene, Tokyo, Japan). The resultant plasmids were designated pET-28b-RS02470 and pET-28b-RS02475. All inserted sequences in the plasmids were confirmed by Sanger sequencing (Eurofins Genomics, Tokyo, Japan).

### Expression and purification of recombinant proteins.

E. coli BL21(DE3) transformed with pET-28b-BH513, pET-28b-RS02470, or pET-28b-RS02475 was grown at 20°C in LB medium containing kanamycin until the culture reached an OD_600_ of 0.5 to 0.7. Protein expression was induced by adding 20 μM isopropyl-β-d-thiogalactoside (IPTG), and incubation was continued overnight at 13°C. The cells were harvested by centrifugation and resuspended in 25 mM Tris-HCl (pH 7.5), 500 mM NaCl, 30 mM imidazole with protease inhibitor cocktail (Nacalai Tesque, Kyoto, Japan). After disruption of the cells by sonication, the lysates were obtained by centrifugation and filtration with a 0.45-μm Minisart syringe filter (Sartorius, Gottingen, Germany). The crude extract was loaded onto an immobilized nickel affinity chromatography column (HisTrap HP; Cytiva, Marlborough, MA) equilibrated with the resuspended buffer. The column was washed with 25 mM Tris-HCl (pH 7.5), 500 mM NaCl, 30 mM imidazole, 5 mM ATP, 10 mM MgCl_2_, and 10% glycerol to remove contaminating proteins, then eluted with a gradient of 30 to 400 mM imidazole.

After concentration of the eluate with an Amicon Ultra-15 centrifugal filter unit (molecular weight cutoff [MWCO], 10,000; Merck Millipore), the concentrated sample was subjected to size exclusion chromatography with a Superdex 200 10/300 GL column (Cytiva), and the fractions that possessed the cell proliferation activity were pooled. The protein concentration was determined with reference to standards of bovine serum albumin using a bicinchoninic acid (BCA) protein assay kit (Fujifilm Wako). The purity of the fractions of each purification step was checked by SDS-PAGE using e-PAGEL HR 10% gel (ATTO, Tokyo, Japan) followed by staining with CBB Stain One Super (Nacalai Tesque). Endotoxin (lipopolysaccharide) levels in the purified protein samples were determined with a Pierce Chromogenic Endotoxin Quant kit (Thermo Fisher Scientific), which is based on the amebocyte lysate method, and confirmed to be <0.1 endotoxin unit (EU) per μg protein. The purified proteins were stored at −80°C until use in each experiment.

### Detection of BafA*_Bba_* from culture supernatant.

Recombinant BafA*_Bba_* passenger domain was used for antiserum production in a rabbit by Eurofins Genomics. Anti-BafA*_Bba_* polyclonal antibody was then purified by using rProtein A Sepharose Fast Flow (Cytiva). For detection of BafA*_Bba_* from culture supernatant, *B. bacilliformis* or B. henselae (3.0 × 10^8^ bacteria/well) was cocultured with HUVECs in 1 mL of M199–0.1% FBS for 48 h in a humidified atmosphere and 5% CO_2_. Each culture was centrifuged to remove the cells, and the supernatants were then concentrated with an Amicon Ultra-15 centrifugal filter unit (MWCO, 10,000). The concentrated supernatants were subjected to SDS-PAGE using Bolt 10% bis-Tris gel and MOPS (morpholinepropanesulfonic acid) SDS running buffer (Thermo Fisher Scientific). The proteins in the gel were transferred onto a polyvinylidene difluoride (PVDF) membrane using an iBlot 2 gel transfer device (Thermo Fisher Scientific). The membrane was then directly stained for total-protein detection using a Revert total-protein staining kit (LI-COR, Lincoln, NE) and subsequently scanned using the 700-nm channel of the Odyssey CLx imaging system (LI-COR). After total-protein detection, the membrane was probed with rabbit anti-BafA*_Bba_* polyclonal antibody followed by IRDye 800CW goat anti-rabbit IgG secondary antibody (LI-COR) using an iBind Western system with an iBind fluorescent detection solution kit (Thermo Fisher Scientific). Finally, the reactive bands were detected using the 800-nm channel of the Odyssey imager.

### Endothelial cell tube formation assay.

To evaluate proangiogenic activity of BafA *in vitro*, an endothelial tube formation assay was performed using collagen gel prepared with a collagen gel culturing kit with concentrated MEM culture solution (Nitta Gelatin, Osaka, Japan) as previously described (19). HUVECs (7 × 10^5^ cells/well in a 48-well plate) were cultured between two layers of collagen gel in the presence of PBS, BafA*_Bhe_* (100 ng/mL), or BafA*_Bba_* (100 ng/mL). Basal EGM with 2% FBS containing PBS, BafA*_Bhe_*, or BafA*_Bba_* was added to the gels in each well, and then 20-h cultures were stained with 1 μM calcein acetoxymethyl (AM) (Dojindo, Kumamoto, Japan). After washing with Hanks’ balanced salt solution (HBSS; Thermo Fisher Scientific), nine fields of fluorescence images in each well were collected using an Opera Phenix system with a 20× objective, followed by creation of global images. From these global images, the total tube area, total tube length, and the numbers of branch points from the tube-like structures were quantified using Harmony 4.5 software.

### Western blot analysis.

For analysis of VEGFR2 and ERK1/2 activation, HUVECs (passage 6 to 9, 10,000 cells/well) were seeded onto gelatin-coated 12-well plates with EGM-2. After 24 h of incubation, the medium was replaced with M199–10% FBS. The cells were starved for 15 h in the same medium and then stimulated with recombinant human VEGF-A_165_ (20 ng/mL), BafA*_Bhe_* (200 ng/mL), or BafA*_Bba_* (200 ng/mL) for the indicated time at 37°C. The cells were harvested, lysed with 0.2 M NaCl, 1 mM EDTA, 1 mM dithiothreitol, protease inhibitor cocktail, and phosphatase inhibitor cocktail (Nacalai Tesque), and sonicated by using a Bioruptor sonicator (Sonicbio, Kanagawa, Japan). After centrifugation to remove cell debris, each cell lysate was then subjected to SDS-PAGE using Bolt 10% bis-Tris gel and MOPS-SDS running buffer. Following electrophoresis, the proteins were transferred onto PVDF membranes using the iBlot 2 gel transfer device.

For subsequent blocking and antibody incubation, iBind Solution kit (Thermo Fisher Scientific) was used according to the manufacturer's instructions. The prepared diluted primary antibodies and horseradish peroxidase (HRP)-conjugated anti-rabbit secondary antibody (1:4,000; number 711-035-152; Jackson ImmunoResearch, West Grove, PA) were sequentially probed with iBind Western system. The primary antibodies (all from Cell Signaling Technology, Danvers, MA) were as follows: VEGFR2 (1:2,000; number 9698), p-VEGFR2 (1:1,000; number 3770), ERK1/2 (1:1,000; number 4695), p-ERK1/2 (1:4,000; number 4370), β-actin (1:4,000; number 4970). The signals were detected with SuperSignal West Femto maximum-sensitivity substrate (Thermo Fisher Scientific) or Chemi-Lumi One Super (Nacalai Tesque) and visualized using LAS-4000 Mini (Fujifilm, Tokyo, Japan). For detection of nonphosphorylated proteins or internal control (β-actin), the blots were stripped with WB stripping solution (Nacalai Tesque) after detection of the phosphorylated protein and reprobed with another antibody. The density of the signals was measured using Multi Gauge version 3.0 (Fujifilm).

### RNA interference.

Silencer Select small interfering RNAs (siRNAs) against VEGFR2 (s7823; Thermo Fisher Scientific) and Silencer Select negative-control siRNA number 2 (Thermo Fisher Scientific) were used for RNAi assays. Briefly, 5,000 cells of HUVEC in EGM-2 were plated in each well of a 96-well plate and incubated at 37°C for 6 h. The medium was replaced with 0.1 mL of M199–10% FBS, and the cells were transfected with siRNA (0.5 pmol/well) using Lipofectamine RNAiMAX (Thermo Fisher Scientific) in accordance with the manufacturer's instructions. After 24 h, the cells were washed and replaced with fresh M199–10% FBS and then used for cell proliferation assays against VEGF-A_165_ and BafA*_Bba_*. VEGFR2 knockdown at 24 h after transfection was confirmed by quantitative reverse transcription-PCR (qRT-PCR).

### qRT-PCR.

Total RNA of HUVECs was purified with RNeasy minikit (Qiagen, Hilden, Germany). cDNA was synthesized with a QuantiTect reverse transcription kit (Qiagen), and subsequent qRT-PCR was performed with TaqMan Fast advanced master mix (Thermo Fisher Scientific) and the desired TaqMan probes (GAPDH, Hs02758991_g1; KDR [VEGFR2], Hs00911700_m1 [Thermo Fisher Scientific]) with a QuantStudio 7 Flex system. The VEGFR2 expression was determined by the ΔΔ*C_T_* method, normalized to GAPDH mRNA expression.

### SPR analysis.

The interaction of recombinant Fc-tagged VEGFR2-ECD (Sino Biological, Beijing, China) with VEGF-A_165_, BafA*_Bhe_*, or BafA*_Bba_* was monitored by SPR using a Biacore 8K (Cytiva) performed at 25°C in single-cycle mode. The VEGFR2-ECD protein (180 nM) was injected and captured on flow cells (Fc) 2 on a series S sensor chip protein A (Cytiva) at approximately 1,400 to 1,700 response units. Fc 1 was used as the negative control. All proteins used for this assay were in HBS-*P*+ buffer (Cytiva). Various concentrations (1.25, 2.5, 5, 10, and 20 nM) of VEGF-A_165_, BafA*_Bhe_*, and BafA*_Bba_* were then flowed through the sensor chip at a flow rate of 30 μL/min and the real-time response was recorded. After each reaction, the sensor chip was regenerated using 10 mM glycine-HCl at pH 1.5. The *K_D_* values were calculated from the first on and off rates from the bivalent model of Biacore Insight Evaluation software (Cytiva).

### Statistical analysis.

Data are expressed as means and standard deviations (SD). Statistical analysis between two groups was performed using two-tailed unpaired Student's *t* test, and statistical significance among multiple groups was analyzed by one-way analysis of variance (ANOVA) with Dunnett’s or Tukey’s multiple-comparison test, as indicated in the figure legends, with GraphPad Prism 9 software (GraphPad Software, La Jolla, CA). All experiments were conducted at least in triplicate to ensure reproducibility of the observations. Representative Western blots and microscopy images from at least three biologically independent replicates with similar results are shown.

### Data availability.

Raw Sanger sequencing data will be provided by the corresponding author on reasonable request. All other data generated during the study are included in this article.
