# Supplementary material for: The Passenger Domain of Bartonella bacilliformis BafA Promotes Endothelial Cell Angiogenesis via the VEGF Receptor Signaling Pathway
Source: mSphere. 2022 Apr 5;7(2):e00081-22. doi: 10.1128/msphere.00081-22 (PMC9044958; doi:10.1128/msphere.00081-22)
Supplement: FIG S1 [file msphere.00081-22-sf001.pdf]

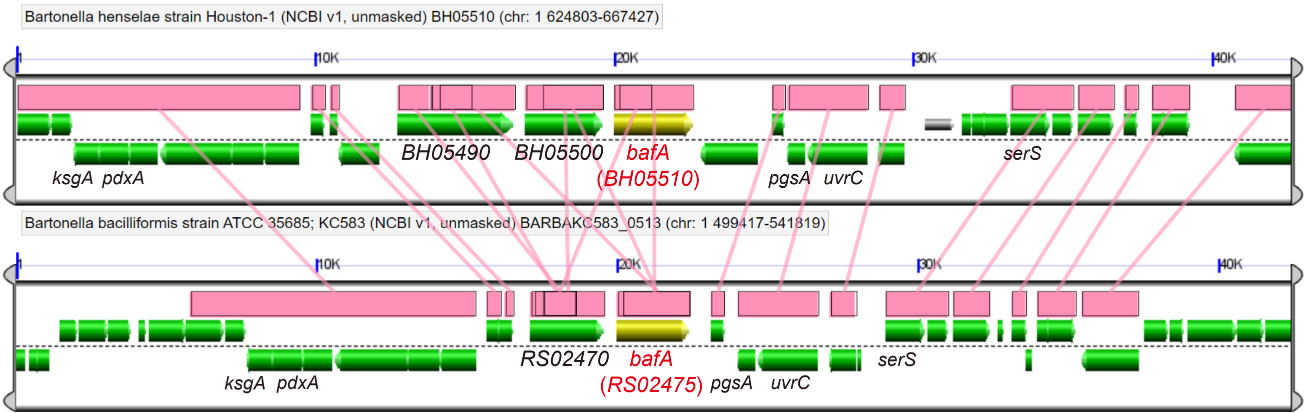

Bartonella henselae strain Houston-1 (NCBI v1, unmasked) BH05510 (chr: 1 624803-667427)
1
10K
20K
30K
40K
BH05490
BH05500
bafA
(BH05510)
serS
ksgA
pdxA
pgsA
uvrC
Bartonella bacilliformis strain ATCC 35685; KC583 (NCBI v1, unmasked) BARBAKC583_0513 (chr: 1 499417-541819)
1
10K
20K
30K
40K
RS02470
bafA
(RS02475)
serS
ksgA
pdxA
pgsA
uvrC
