# Supplementary material for: The Passenger Domain of Bartonella bacilliformis BafA Promotes Endothelial Cell Angiogenesis via the VEGF Receptor Signaling Pathway
Source: mSphere. 2022 Apr 5;7(2):e00081-22. doi: 10.1128/msphere.00081-22 (PMC9044958; doi:10.1128/msphere.00081-22)
Supplement: TEXT S1 [file msphere.00081-22-s0001.docx]

**Text S1: Supplemental Methods**

**Bacterial culture.** *Bartonella henselae strain* strain Houston-1 (ATCC 49882) was obtained from the American Type Culture Collection. *B. henselae* was grown on Columbia agar with 5% defibrinated sheep blood (CSB) at 35°C for 3 to 7 days in a humidified atmosphere and 5% CO_2_. *B. henselae* mutant with transposon disruption in *bafA* (clone 623-125) and its complemented strain were generated in our previous study (1). *Escherichia coli* S17-1 λ-pir was obtained from National BioResource Project (NIG, Japan): *E. coli*, and grown on a lysogenic broth (LB) agar or in the liquid medium. When required, kanamycin (25 µg/mL), ampicillin (100 µg/mL), ofloxacin (0.5 µg/mL), or trimethoprim (10 µg/mL) was used.

**Plasmid construction.** For complementation of *bafA_Bba_* in the *bafA*-disrupted mutant of *B. henselae*, the entire *BARBAKC583_RS02475* gene was amplified by PCR with primers BB2475-Fw (ATGAAAAATGAATATAGATTGGTTTTTTG) and BB2475-Rv (GCCTTAGCTCCTGATATCCTAAAAACTATAACGCAAACCAATTAAA) using the *B. bacilliformis* genomic DNA as the template. In addition, the region including putative promotor of *bafA_Bhe_* was amplified with primers BheP-Fw (TAGAACTAGTGGATCCGTTTATACGTTTATTTTCTGGTCAAT) and BheP-Rv (ATATTCATTTTTCATGATTCACACTCCACTATCAATAA). These two PCR products were inserted into the BamHI/EcoRV sites of pBBR1-TpR, the broad host range expression vector with a trimethoprim resistance gene (1), using In-Fusion HD cloning kit (Takara Bio Inc., Shiga, Japan). Finally, the ampicillin resistance gene (*bla*) amplified from pMariK vector (1) with primers bla-Fw (AGCAAACTGGCCTCACGGAGTGTATACTGGCTTAAC) and bla-Rv (TTCTCAAATGCCTGA CACTTTTCGGGGAAATGTG) was inserted into the Bsu36I site of the resultant plasmid in the same manner. The generated plasmid was designated pBAFBba. The inserted sequences in the plasmid were confirmed by Sanger sequencing.

**Biparental conjugation.** The donor *E. coli* S17-1 λ-pir was transformed with pBAFBba and grown overnight in LB medium containing ampicillin. The culture was washed with 10 mM MgSO_4_ once and resuspend in 1 mL of heart infusion broth (Becton Dickinson) to an optical density at 600 nm (OD_600_) of 0.1. The recipient *B. henselae* 623-125 was grown on CSB for 4 days, collected from plates, and suspended with 1 mL of heart infusion broth to an OD_600_ of 10. The suspensions of *E. coli* and *B. henselae* were mixed at a ratio of 1:10 and dropped onto CSB. After drying for 30 minutes, the plate was incubated for 7 hours at 37°C in a humidified atmosphere and 5% CO_2_. The conjugated bacteria were collected, plated onto CSB containing 0.5 µg/mL of ofloxacin, and incubated for 16 hours to remove the donor *E. coli*. Subsequently, the bacterial cells were harvested from the plate, suspended and spread onto CSB containing ofloxacin, kanamycin, and trimethoprim. After 14 days of culture, the colony of pBAFBba-introduced *B. henselae* was obtained.

**Synteny analysis.** Comparison of the genomic regions containing *bafA* orthologs was performed by GEvo in CoGe (https://genomevolution.org/coge/GEvo.pl). The 43-kb regions containing each *bafA_Bhe_* (CoGe data base name: BH05510) or *bafA_Bba_* (BARBAKC583_0513) were submitted to GEvo using the ‘(B)LastZ: Large Regions’ algorithm with a score threshold of 3,000.

**Reference**

1. Tsukamoto K, Shinzawa N, Kawai A, Suzuki M, Kidoya H, Takakura N, Yamaguchi H, Kameyama T, Inagaki H, Kurahashi H, Horiguchi Y, Doi Y. 2020. The *Bartonella* autotransporter BafA activates the host VEGF pathway to drive angiogenesis. Nat Commun 11:3571.
